# Impact of human mesenchymal stromal cells on antifungal host response against *Aspergillus fumigatus*

**DOI:** 10.18632/oncotarget.20753

**Published:** 2017-09-08

**Authors:** Stanislaw Schmidt, Lars Tramsen, Andreas Schneider, Ralf Schubert, Ada Balan, Özer Degistirici, Roland Meisel, Thomas Lehrnbecher

**Affiliations:** ^1^ Divisions for Pediatric Hematology and Oncology, Hospital for Children and Adolescents, Johann Wolfgang Goethe-University, Frankfurt, Germany; ^2^ Divisions for Pediatric Pulmonology, Allergology and Cystic Fibrosis, Hospital for Children and Adolescents, Johann Wolfgang Goethe-University, Frankfurt, Germany; ^3^ Division for “Victor Babes”, University of Medicine and Pharmacy, Timisoara, Romania; ^4^ Division of Pediatric Stem Cell Therapy, Clinic for Pediatric Oncology, Hematology and Clinical Immunology, Medical Faculty, Heinrich-Heine-University, Düsseldorf, Germany

**Keywords:** mesenchymal stromal cells, Aspergillus fumigatus, phagocytosis, hematopoietic stem cell transplantation, immunotherapy

## Abstract

Mesenchymal stromal cells (MSCs) are increasingly given as immunotherapy to hematopoietic stem cell transplant (HSCT) recipients with refractory graft-versus-host disease (GvHD). Whereas the immunosuppressive properties of MSCs seem to be beneficial in GvHD, there is, at the same time, major concern that MSCs increase the risk for infection. We therefore investigated the interplay of human MSCs with *Aspergillus fumigatus* and the impact of MSCs on different arms of the anti-*Aspergillus* host response *in vitro*. Although *A. fumigatus* hyphae increase mRNA levels of *IL6* in MSCs, the extracellular availability of IL-6 and other pro-inflammatory cytokines remains unaffected. Human MSCs are able to phagocyte *Aspergillus* conidia, but phagocytosis of conidia is not associated with an alteration of the cytokine production by MSCs. In addition, human MSCs do not affect activation and function of *A. fumigatus* specific CD4^+^ T cells, and MSCs do not negatively impact the oxidative burst activity of phagocytes. Our *in vitro* data indicate that administration of human MSCs is not associated with a negative impact on the host response against *A. fumigatus* and that the fungus does not stimulate MSCs to increase the release of those cytokines which play a central role in the pathophysiology of GvHD.

## INTRODUCTION

Patients undergoing allogeneic hematopoietic stem cell transplantation (HSCT) and suffering from graft-versus-host disease (GvHD) are at a significantly increased risk for invasive aspergillosis [1]. Whereas the standard intervention in GvHD consists of immunosuppressive drugs, there is growing interest in cellular therapy with mesenchymal stromal cells (MSCs) in patients refractory to first-line treatment [2]. Mesenchymal stromal cells possess multi-lineage differentiation potential and extensive immunomodulatory properties towards both innate and adaptive immune system by the release of soluble factors including interferons (IFN) and interleukins (IL) [3, 4]. Although MSCs seem to be a promising strategy in GvHD, their immunosuppressive properties raised concern that MSCs may inadvertently inhibit antimicrobial immune responses, and thus further increase the risk for infection. At the same time, MSCs have specific antimicrobial and pro-inflammatory effects, which might aggravate GvHD during an infectious episode [4]. Unfortunately, clinical studies in large and homogenous patient populations on the impact of adoptively transferred MSCs on infectious complications are difficult to perform. We therefore thought to perform *in vitro* studies evaluating the interplay of MSCs with *Aspergillus fumigatus* and the impact of MSCs on different arms of the anti-*Aspergillus* host response.

## RESULTS

### Impact of A. fumigatus hyphae and conidia on the gene expression and extracellular availability of selected pro- and anti-inflammatory cytokines in human MSCs

**Figure 1:**
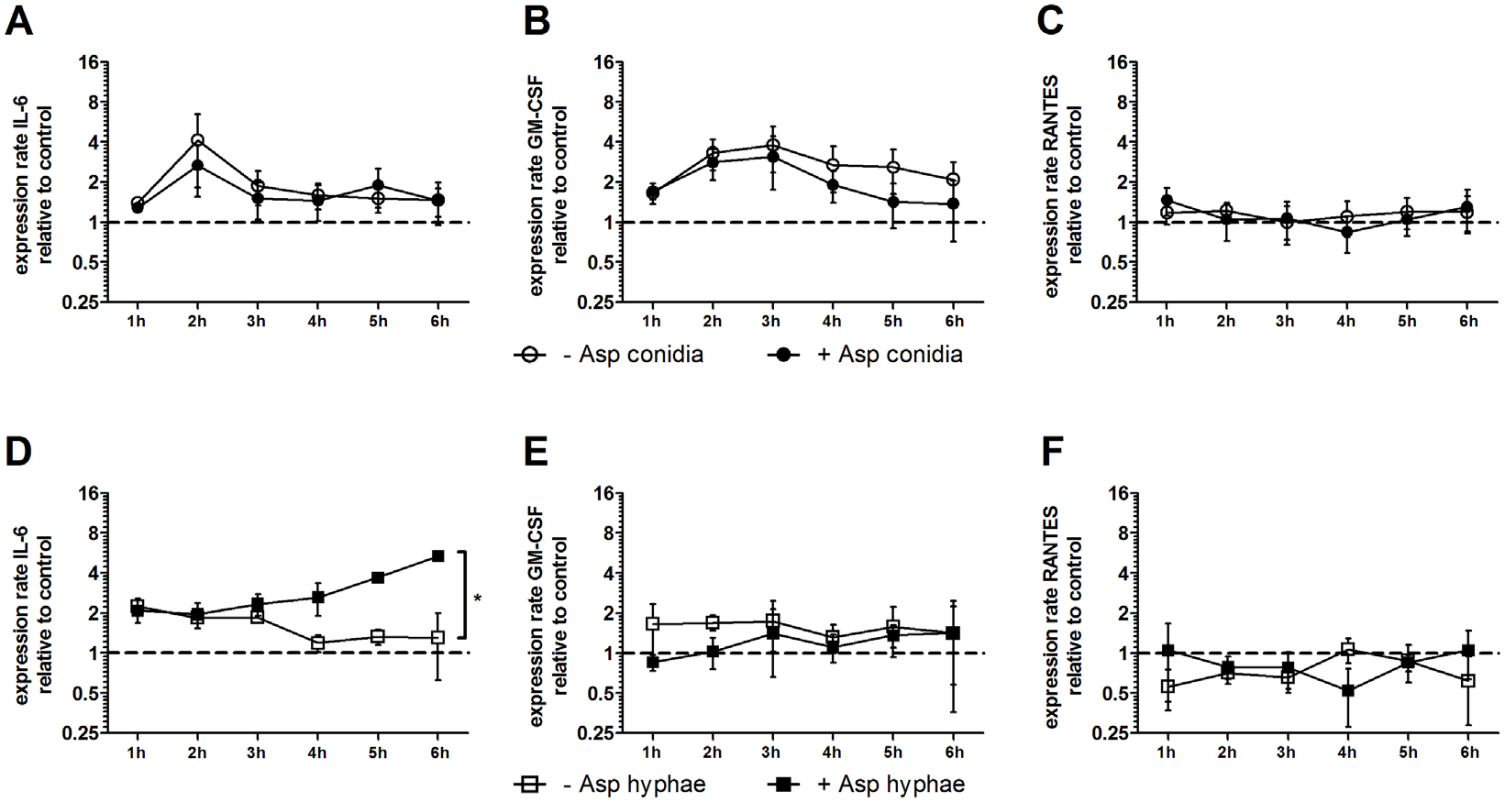
Effect of *A. fumigatus* conidia **(A-C)** and hyphae **(D-F)** on the gene expression of *IL6*, *RANTES* and *GMCSF* in mesenchymal stromal cells (MSCs). Gene expression of *IL6*, *RANTES* and *GMCSF* in human MSCs co-incubated with *A. fumigatus* conidia (filled dots) or *A. fumigatus* hyphae (filled squares) or incubated alone (open dots/squares). The X axis represents the time (hours); the first assessment of transcript levels was performed at hour 1. The Y axis represents the relative fold-change of gene expression at specific time points to gene expression at time point 0 (dotted line; <1 down-regulation, >1 up-regulation). Squares and dots represent means, bars the standard error of means (n=3). The *P* value represents the difference at time point 6 hours. ^*^P < 0.01

**Figure 2:**
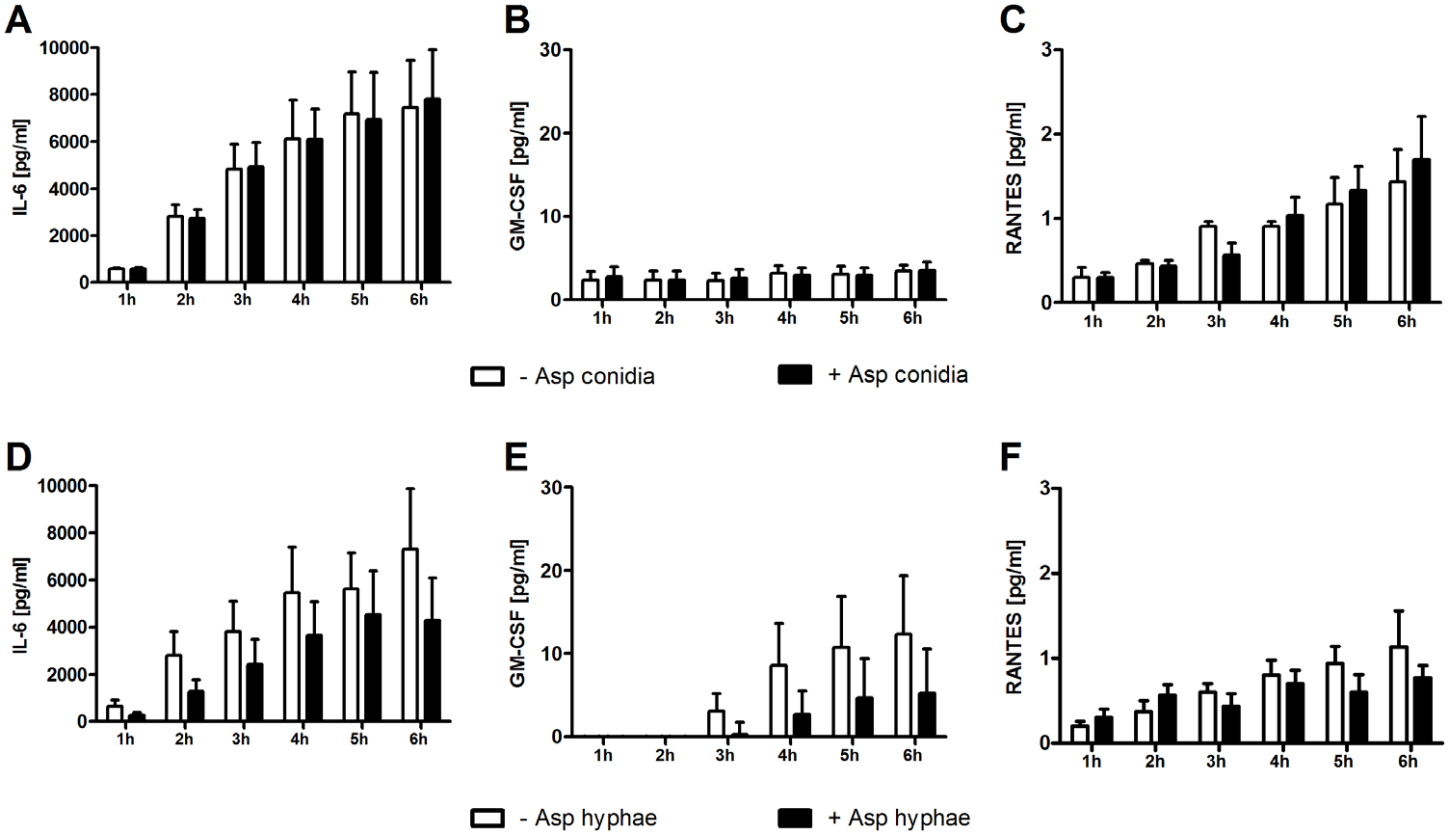
Effect of *A. fumigatus* conidia **(A-C)** and hyphae **(D-F)** on the cytokine concentration in the supernatant of mesenchymal stromal cells. Shown are mean and SEM from three independent experiments.

As pro- and anti-inflammatory cytokines such as IFN-γ, TNF-α, GM-CSF, RANTES, IL-17, IL-4, and IL-10 have an important impact on the antifungal host response, as well as cytokines such as IL-6 play a central role in the pathophysiology of GvHD, we assessed the gene expression and extracellular concentration of these molecules in MSCs which were co-incubated with or without *A. fumigatus* conidia and hyphae, respectively [5, 6]. When co-incubated with *A. fumigatus* conidia, the gene expression of *IL6, RANTES* and *GMCSF* in human MSCs was not significantly altered (Figure 1A-1C). Similarly, the concentration of these molecules in the supernatant was comparable in the presence or absence of *A. fumigatus* conidia (Figure 2A-2C).

In contrast, when co-incubated with *A. fumigatus* hyphae, mRNA levels of *IL6* increased in human MSCs by 4-fold as compared to MSCs incubated alone (mean x-fold change±SEM, 1.3±0.7 vs. 5.3±0.4, *P*<0.01 after 6 hours), whereas the gene expression of *RANTES* and *GMCSF* was not affected (Figure 1D-1F). However, the protein levels of all molecules measured in the supernatant after 6 hours decreased by co-incubation with *A. fumigatus* hyphae compared to MSCs incubated alone, although this decrease did not reach statistical difference (Figure 2D-2F). Levels of mRNA and protein of both IL-17 and the anti-inflammatory cytokines IL-4 and IL-10 were not detectable.

### Human MSCs are able to phagocyte *A. fumigatus* conidia

**Figure 3:**
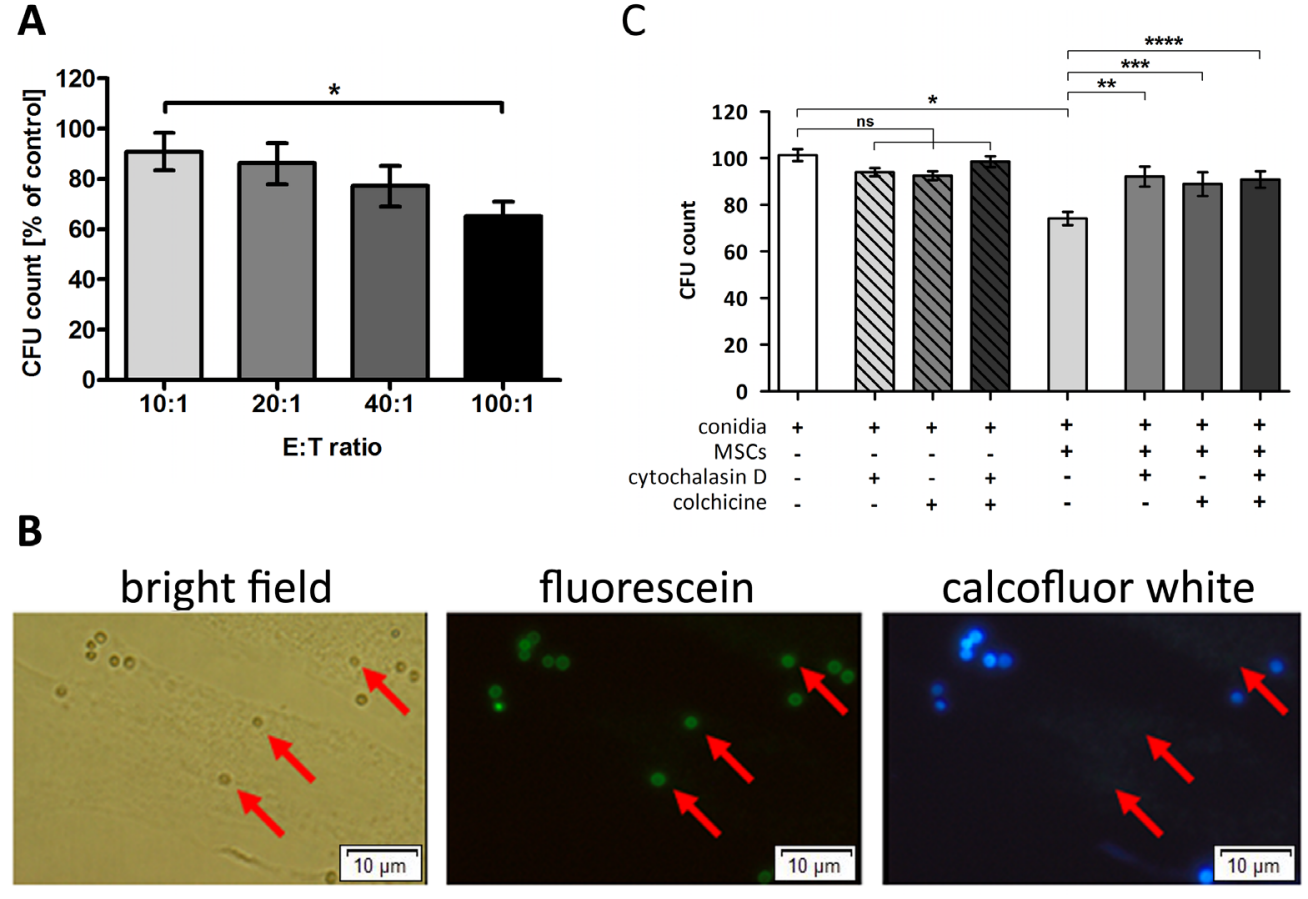
Human mesenchymal stromal cells (MSCs) are able to phagocyte *Aspergillus fumigatus* conidia **(A)** Co-incubation of resting conidia of *A. fumigatus* with human MSCs resulted in a decreased number of colony forming units (CFU) in an effector:target (E:T) ratio dependent manner. Conidia incubated alone served as control (100%). The bars represent mean, the whiskers SEM of four independent experiments, each of them performed in duplicates; ^*^P <.05. **(B)** FITC-prelabeled *A. fumigatus* conidia were incubated with human MSCs for 4 h, and calcofluor white staining was performed subsequently. Extracellular located conidia fluoresced with FITC and were also counterstained with calcofluor white, while intracellular conidia (indicated by red arrows) only retained a green signal resulting from FITC pre-labeling. The picture displays one representative experiment out of a total of three experiments (magnification 40x). **(C)** *A. fumigatus* conidia were incubated with human MSCs at an E:T ratio of 40:1 for 4 h. Phagocytosis inhibitors cytochalasin D (1 μmol/L) and colchicine (2 μmol/L) were added to the experiment, individually or in combination. Co-incubation of *A. fumigatus* resting conidia with untreated MSCs significantly reduced the number of CFUs, while the addition of the phagocytosis inhibitors almost completely reversed the effect. Conidia incubated alone served as control. The bars represent mean, the whiskers SEM of three independent experiments, each of them performed in duplicates; ns = not significant, ^*^P <.0001, ^**^P <.005, ^***^P <.05, ^****^P <.001.

Co-incubation of *A. fumigatus* conidia with human MSCs resulted in a dose dependent reduction of the formation of fungal colonies (Figure 3A). This effect was not seen when the supernatant of MSCs alone was added to the fungus (data not shown), suggesting that cellular mechanisms play a role in the antifungal activity. When co-incubating FITC pre-labeled conidia with MSCs, all conidia were detectable by fluorescence microscopy in both bright field and fluorescein channel, whereas only part of the conidia were detected by calcofluor white staining (Figure 3B), indicating that these conidia were located intracellularly in the MSCs. When adding colchicine and cytochalasin D to block phagocytosis, the effect of MSCs on colony formation was almost completely abrogated (Figure 3C).

### Impact of human MSCs on human anti-*Aspergillus* T cells

**Figure 4:**
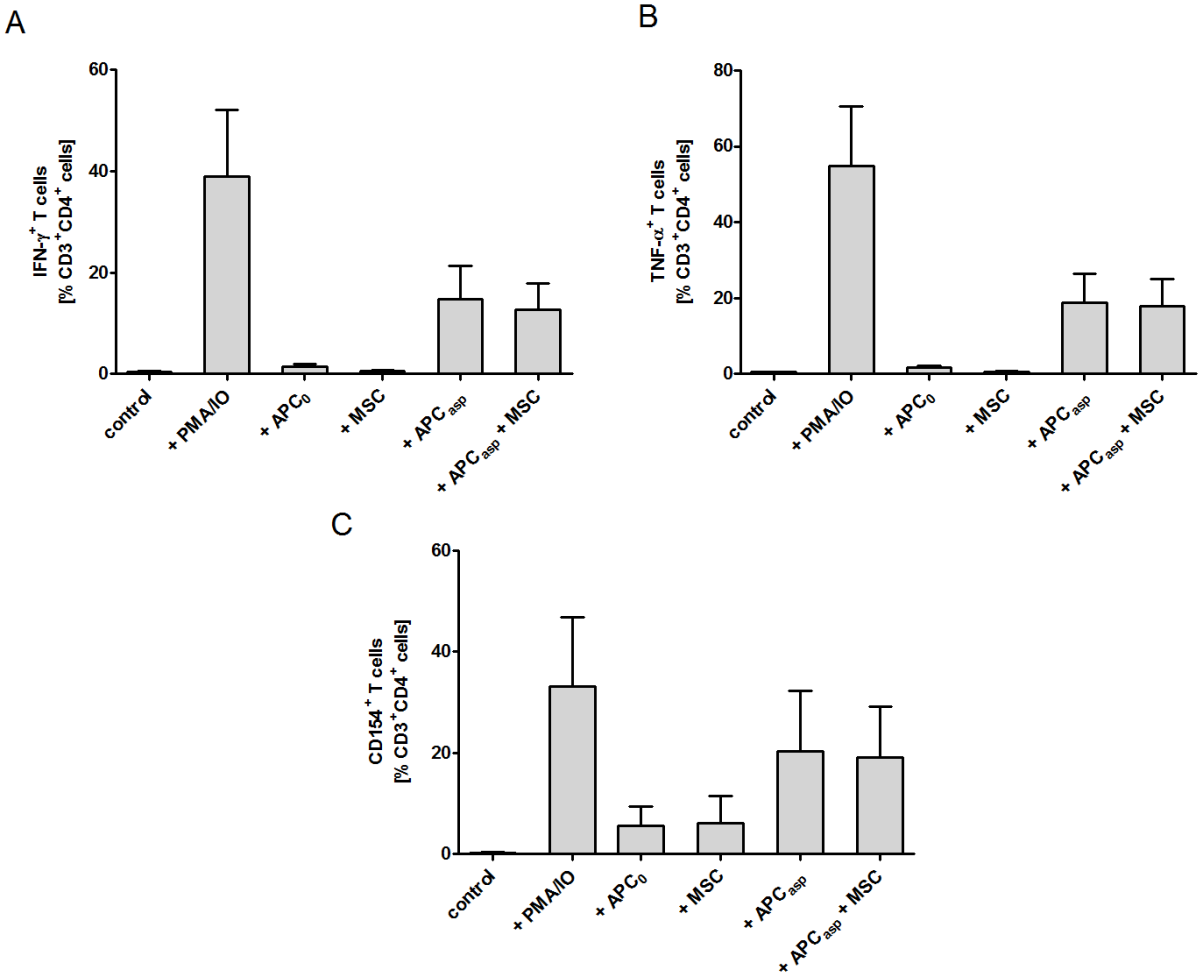
Human mesenchymal stromal cells (MSCs) do not alter the percentage of IFN-γ and TNF-α producing and CD154^+^ *Aspergillus*-specific T_H_1 cells Unstimulated *Aspergillus*-specific T cells served as negative, and PMA stimulated T cells as positive control. The bars represent mean, the whiskers SEM of three independent experiments. APC_0_ unstimulated antigen presenting cell; APC_Asp_ *A. fumigatus* antigen-stimulated antigen presenting cell.

As T cells play a major role in both the pathogenesis of GvHD and the antifungal host response, we investigated whether human MSCs have an impact on activation and function of human anti-*Aspergillus* T cells. When co-incubating anti-*Aspergillus* CD4^+^ T cells with *Aspergillus* antigens-loaded antigen presenting cells (APCs) or unloaded APCs, the percentage of activated and CD154 expressing T cells as well as the percentage of IFN-γ^+^ and TNF-α^+^ secreting T cells among anti-*Aspergillus* T cells was higher in the presence of *Aspergillus* antigens-loaded APCs as compared to unloaded [CD154 (mean±SEM, 20.3±11.9% vs. 5.6±3.8%), IFN-γ (mean±SEM, 14.7±6.6% vs. 1.5±0.5%), TNF-α (mean±SEM, 18.9±7.6% vs. 1.8±0.4%), Figure 4]. However, no significant effect was observed when MSCs were added to either setting (Figure 4).

**Figure 5:**
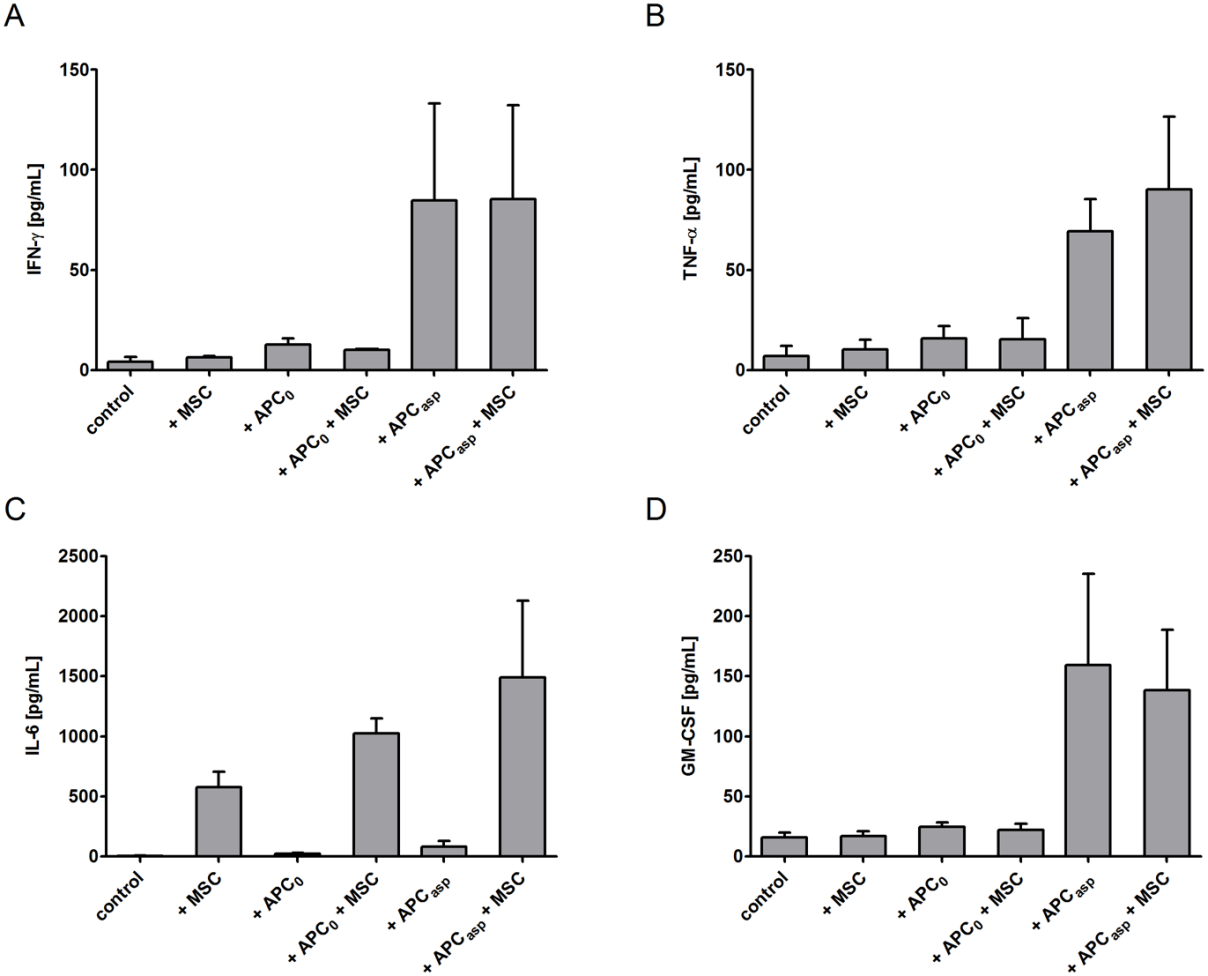
Impact of human mesenchymal stromal cells (MSCs) on the concentration of cytokines in the supernatant of human anti-*Aspergillus* T cells Anti-*Aspergillus* CD4^+^ T cells incubated alone served as control. Shown are mean and SEM from three independent experiments. APC_0_ unstimulated antigen presenting cell; APC_Asp_ *A. fumigatus* antigen-stimulated antigen presenting cell.

When assessing the cytokine levels in the supernatant, adding human MSCs increased the concentrations of IL-6 of anti-*Aspergillus* T cells incubated with *Aspergillus* antigens-pulsed APCs (mean±SEM, 1488.3±640.8 pg/mL vs. 79.8±48.6 pg/mL) as well as of anti-*Aspergillus* T cells co-incubated with unloaded APCs alone (1024±123.8 pg/mL vs. 21.4±8.7 pg/mL). However, IL-6 levels were also increased in the supernatant of anti-*Aspergillus* T cells incubated with human MSCs alone as compared to the control (mean±SEM, 576.6±127.7 pg/mL vs. 4.5±2.7 pg/mL) (Figure 5C). In contrast, adding MSCs to anti-*Aspergillus* T cells co-incubated with *Aspergillus* antigens-loaded APCs did not significantly alter the levels of IFN-γ (mean±SEM, 84.7±48.4 pg/mL vs. 85.3±47.1 pg/mL, Figure 5A), TNF-α (mean±SEM, 69.2±16.2 pg/mL vs. 90.2±36.3 pg/mL, B) and GM-CSF (mean±SEM, 159.3±75.9 pg/mL vs. 138.2±50.4 pg/mL, D) in the supernatant. Similarly, comparable concentrations of the respective cytokines in the supernatant were observed when anti-*Aspergillus* T cells were co-incubated with unloaded APCs, with human MSCs, or with both unloaded APCs and MSCs, respectively (mean±SEM, IFN-γ: 12.6±3.1 pg/mL, 6.2±0.8 pg/mL, 10.2±0.3 pg/mL, Figure 5A; TNF-α: 15.7±6.2 pg/mL, 10.3±4.7 pg/mL, 15.3±10.7 pg/mL, 5B; GM-CSF: 24.4±3.9 pg/mL, 16.7±4.1 pg/mL, 21.7±5.4 pg/mL, 5D).

No differences were seen in any setting when human MSCs were pre-stimulated with exogenous IFN-γ prior to the experiment (data not shown).

### Impact of human MSCs on professional phagocytes

**Figure 6:**
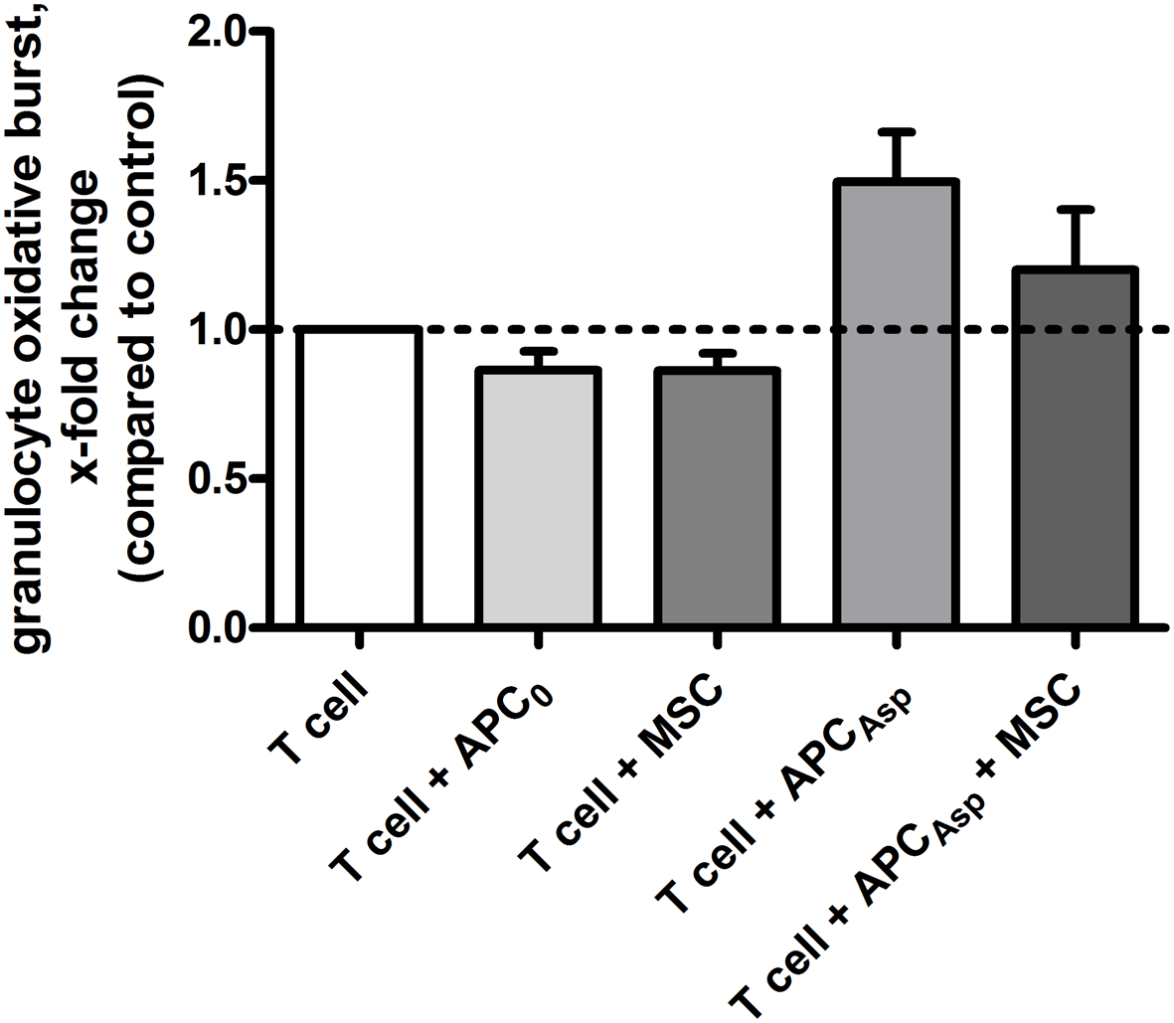
Human mesenchymal stromal cells do not alter the effect of unstimulated or stimulated human anti-*Aspergillus* T cells on oxidative burst activity of granulocytes Shown are mean and SEM of the relative change in oxidative burst activity of granulocytes compared to the activity elicited by the supernatant of unstimulated human anti-*Aspergillus* T cells (horizontal dashed line). The results of four independent experiments are shown. APC_0_ unstimulated antigen presenting cell; APC_Asp_ *A. fumigatus* antigen-stimulated antigen presenting cell.

As phagocytes play a major role in the first-line host response against *A. fumigatus*, we investigated the effect of MSCs on the oxidative burst activity of granulocytes. We observed that, as compared to the supernatant of an unstimulated T cell control, the supernatant of human anti-*Aspergillus* T cells co-incubated with *Aspergillus* antigens-loaded APCs increased the oxidative burst activity of granulocytes by 1.5±0.2 (mean x-fold change±SEM) (Figure 6). The increase of the oxidative burst activity of granulocytes was not significantly altered when the supernatant of anti-*Aspergillus* T cells antigens-loaded APCs co-incubated with human MSCs was used (1.2±0.2). As compared to the unstimulated T cell control, a similar oxidative burst activity of granulocytes was seen when using the supernatant of human MSCs alone or of human anti-*Aspergillus* T cells co-incubated with unloaded APCs alone (each 0.9±0.1) (Figure 6).

## DISCUSSION

Mesenchymal stromal cells (MSCs) are increasingly administered to patients suffering from GvHD refractory to first-line treatment [2]. However, the immunosuppressive properties of MSCs raised concern whether this strategy further increases the high risk for IFD in this patient population. As no conclusive data of clinical trials are available, and studies with large and homogenous patient populations are difficult to perform, we thought to investigate the interplay of human MSCs and *A. fumigatus* as well as the effects of human MSCs on the antifungal host response *in vitro*.

In a first step, we co-incubated *A. fumigatus* hyphae with human MSCs and observed a significantly increased gene expression of *IL6* in MSCs, whereas the protein level measured in the supernatant considerably decreased, although this decrease did not reach statistical significance. A similar finding with decreased extracellular availability of the protein despite increased mRNA levels was recently reported for NK cell -derived IFN-γ, which was due to a decreased release of IFN-γ when NK cells were co-incubated with *A. fumigatus* hyphae [7]. In contrast to *IL6*, co-incubation with *A. fumigatus* hyphae did not significantly alter the gene expression of other pro-inflammatory molecules such as RANTES and GM-CSF as well as of anti-inflammatory molecules such as IL-4, IL-10, and IL-17A.

In contrast to *A. fumigatus* hyphae, conidia of the fungus did not affect the gene expression and protein release of any of the investigated molecules, corroborating previous results in other immune cells such as T cells or NK cells, where co-incubation with *Aspergillus* conidia did not result in any effect. However, our data indicate that human MSCs are able to phagocyte *A. fumigatus* conidia, which has not described before. As conidia are the resting and not the invasive form of *Aspergillus* spp., further research needs to evaluate whether this observation reflects a relevant host response mechanism *in vivo*. To date, data on antifungal properties of MSCs have only been reported in the mouse, where murine MSCs inhibit the growth of *Candida albicans* directly by IL-17 [8]. This is in contrast to our data observed in human MSCs co-incubated with *A. fumigatus*, where we did not find relevant levels of IL-17 mRNA and protein, and where the supernatant of MSCs did not exhibit activity against *Aspergillus* conidia. Unfortunately, due to technical reasons, we were not able to investigate the direct antifungal effect of human MSCs against *A. fumigatus* hyphae.

As human MSCs exhibit important immuno-regulatory mechanisms, we investigated the effect of MSCs on important arms of the antifungal host response, such as on anti-*Aspergillus* CD4^+^ T cells or on phagocytes. Although neutropenia is the most important single risk factor for invasive aspergillosis, there is a growing body of evidence that also T cells play a critical role in the antifungal host response. For example, a proof-of principle study demonstrated that the adoptive transfer of anti-*Aspergillus* T cells in haploidentical transplant recipients with proven or probable aspergillosis improved the prognosis as compared to patients not receiving anti-*Aspergillus* T cells [9]. Our data show that human MSCs do not affect the activation or the functional properties of anti-*Aspergillus* CD4^+^ T cells assessed by CD154 or by the production of IFN-γ and TNF-α. Similar results were observed with unstimulated and IFN-γ pre-activated MSCs, both of which are used in the clinical setting for the prevention or treatment of GvHD [10]. In contrast to the levels of IFN-γ, TNF-α, RANTES, and GM-CSF in the supernatant, all of which were unaffected by MSCs, adding MSCs to anti-*Aspergillus* T cells co-incubated with *Aspergillus*-antigens loaded APCs increased the concentration of IL-6. As human MSCs incubated alone demonstrated considerably higher IL-6 production than anti-*Aspergillus* T cells co-incubated with *Aspergillus*-antigens loaded APCs or with unloaded APCs, respectively, it seems more likely that anti-*Aspergillus* T cells enhance the IL-6 production of MSCs rather than MSCs stimulate IL-6 production by anti-*Aspergillus* T cells. Our findings are in contrast to a recent study which reported that MSCs reduced the percentage of CD4^+^ and CD8^+^ T cells producing TNF-α, IFN-γ and IL-2 in all functional compartments as well as the amount of cytokines produced [11]. The different observations may be explained by the fact that the authors of this study stimulated the cells with PMA and ionomycin, whereas we used *Aspergillus* antigens-loaded APCs as they reflect more the *in vivo* situation. In addition, we employed highly purified cell populations, such as antigen-specific anti-*Aspergillus* CD4^+^ T cells, whereas in experiments in which peripheral blood mononuclear cells were used, the presence of other cells such as regulatory T cells might have a significant impact on the results [12]. In addition, the immunomodulatory effect of MSCs might depend on the developmental stage of the T cell [10, 13].

Our data further demonstrate that human MSCs do not impair the oxidative burst activity of neutrophils, which play a crucial role in the early host defense against fungi [14]. Interestingly, in the murine system, MSCs-treated macrophages significantly reduced *A. fumigatus* conidia growth as compared to macrophages alone, but the mechanism of this effect remained unclear [15].

In conclusion, our data demonstrate that co-incubation of human MSCs with *A. fumigatus* hyphae or conidia do not significantly alter the levels of pro-inflammatory cytokines in the supernatant. In addition, human MSCs do not affect activation and function of *A. fumigatus* specific CD4^+^ T cells, and human MSCs do not negatively impact the oxidative burst activity of phagocytes. The observation that human MSCs are able to phagocyte *Aspergillus* conidia merits further investigation. Our *in vitro* data indicate that administration of human MSCs is not associated with a negative impact on the host response against *A. fumigatus* and that the fungus does not stimulate MSCs to increase the release of those cytokines which play a central role in the pathophysiology of GvHD.

## MATERIALS AND METHODS

### Aspergillus conidia and hyphae

Conidia from the *Aspergillus fumigatus* strain AF4215, which was kindly provided by Cornelia Lass-Flörl, Innsbruck, Austria, were prepared as previously described [16]. For the generation of hyphae, conidia were plated in 48-well flat-bottom cell culture plates (Nunc, Langenselbold, Germany) and incubated in Yeast Nitrogen Base (Sigma-Aldrich, Taufkirchen, Germany) supplemented with (D)-Glucose (Sigma-Aldrich) at 37°C for 17 hours to allow formation of mycelium. For some experiments, conidia were labeled with fluorescein isothiocyanate (FITC)[17] prior to co-incubation with human MSCs. Therefore conidia in 0.1 M carbonate buffer of pH 9.0 were incubated with FITC at a final concentration of 0.1 mg/mL overnight with rotating at 4 °C. To discriminate between attached and ingested conidia, cells and conidia were washed with phosphate buffered saline (PBS) and stained with calcofluor white (Sigma-Aldrich) as previously described [18].

### Mesenchymal stromal cells (MSCs)

Primary human MSCs of healthy donors were generated and prepared as previously described [19]. At a confluence of 80-90%, MSCs were harvested by adding TrypLE Express (Gibco, Paisley, UK). The cell number was determined by trypan-blue stain in a Neubauer-chamber (LO–Laboroptik, Friedrichsdorf, Germany). The study was approved by the Ethics committee (Votum 1830).

### Anti-Aspergillus activity of human MSCs

For assessing the anti-*Aspergillus* activity, MSCs were incubated in 48-well-plates (Nunc) overnight. After washing, cells were co-incubated with 1000 conidia in 1 mL medium at different effector:target ratios. After 4 hours, 100 μL were spread on a Sabouraud glucose agar plate (Becton Dickinson, Heidelberg Germany) and incubated overnight. The anti-conidial effect was assessed by comparing the average number of colony-forming units (CFUs) with and without co-incubated MSCs, respectively. Cytochalasin D (1 μmol/L) and colchicine (2 μmol/L) (Sigma-Aldrich) were used as phagocytosis inhibitors as described previously [20].

### Preparation of RNA and gene expression analysis

MSCs were co-incubated alone or with *Aspergillus* conidia or hyphae, respectively, in 48-well flat-bottom cell culture plates (Nunc) for up to 6 hours. At every hour beginning at hour 1, MSCs from one well of each setting were lysed and total RNA (RNeasy Plus Mikro Kit, QIAGEN, Hilden, Germany) was extracted. Quantitative real-time PCR (qRT-PCR) was performed with cDNA (High-Capacity RNA-to-cDNA Kit, Invitrogen, Darmstadt, Germany) from total RNA using Taqman-probes (Universal ProbeLibrary Technology, Roche, Mannheim, Germany) according to the manufacturers´ instructions and using iQ5 cycler and software version 2.0 (BioRad, München, Germany). Glyceraldehyde 3-phosphate dehydrogenase (GAPDH) served as reference gene. The gene expression analysis was performed by the 2^-ΔΔCt^-method [21].

### Generation of highly purified human anti-Aspergillus CD4^+^ T cells

Anti*-Aspergillus* CD4^+^ T cells were isolated from peripheral blood mononuclear cells (PBMCs) of healthy individuals as previously described [22]. A total of 1×10^8^ PBMCs obtained by density-gradient centrifugation (Biochrome, Berlin, Germany) were added at a concentration of 1×10^7^/mL to cytotoxic T lymphocyte (CTL) medium containing RPMI1640 (Gibco), 100 IU/mL penicillin G, 100 μg/mL streptomycin (Gibco), and 10% pooled heat-inactivated human serum and stimulated for 16 h with 7.5 μg/mL *Aspergillus fumigatus* cell extract (water soluble cellular extracts of *A. fumigatus* (CBS 144-89) [23]). Activated T cells were isolated using the CD154 MicroBead Kit according to the manufacturer´s instructions (Miltenyi Biotec, Bergisch Gladbach, Germany). Enriched cells were then stimulated with 50 IU/ml recombinant human interleukin (rhIL)-2 (Chiron, Ratingen, Germany) every other day for up to 14 days and with 2.5 × 10^5^ irradiated autologous *A. fumigatus*-loaded Antigen-presenting cells (APCs) on days 4, 7 and 10. APCs were obtained by the adherence method and were loaded with the respective antigens (7.5 μg/mL) over night. Thereafter, IFN-γ-secreting cells were enriched using the IFN-γ secretion assay (Miltenyi Biotec) and expanded using a rapid expansion protocol for human T cells [22].

Phenotyping of purified T-cells was performed by an 8-color flow-cytometer (FACS Canto II; BD Biosciences, San Jose, USA). Assessment of intracellular cytokine secretion was performed by means of intracellular cytokine flow cytometry (ICC) as described previously [22, 24]. Positive controls were performed by stimulation with phorbol 12-myristate 13-acetate (PMA; 0.5 μg/mL; Sigma-Aldrich) and ionomycin (1 μg/mL; Sigma-Aldrich).

### Assessment of oxidative burst activity

The effect of anti-*Aspergillus* T cells on professional phagocytes such as granulocytes and monocytes was assessed by means of the Bursttest (Phagoburst®, Orpegen Pharma, Heidelberg, Germany), which was used according to the manufacturer´s instructions with some modifications. In brief, 100 μL of supernatant of anti-*Aspergillus* T cells, which had been pre-incubated with *Aspergillus* antigens-loaded APCs, with unstimulated APCs, or alone, in the presence or absence of human MSCs, respectively, were added to 100 μl fresh blood. As negative and positive controls, culture medium or culture medium supplemented with PMA or fMLP (*N*-formyl-met-leu-phe) was added, respectively. After incubation for 10 min, the fluorogen substrate dihydrorhodamin 123 was added and the turnover of the substrate, indicating the oxidative burst activity of the cells, was analyzed by means of flow cytometry (FACS Canto II, BD Biosciences).

### Assessment of protein concentrations

Pro- and anti-inflammatory cytokines were selected by their reported role in the antifungal host response or in the pathophysiology of GvHD, respectively [5, 6]. The concentrations of cytokines and soluble molecules were assessed using the cytokine bead array (CBA; BD Biosciences) according to the manufacturer´s instructions. The lower limits of detection were 1.4 pg/mL for IL-4, 2.5 pg/mL for IL-6, 0.13 pg/mL for IL-10, 0.3 pg/mL for IL-17, 0.8 pg/mL for IFN-γ, 0.2 pg/mL for GM-CSF, 0.1 pg/mL for CCL5/RANTES and 0.7 pg/mL for TNF-α.

### Microscopy

The fluorescence microscopy was performed using Olympus IX71 microscope system with Olympus LUCPlanFLN 40x objective, with attached Olympus F-View Soft Imaging System camera using the CellSens acquisition software version 1.3 (all Olympus Corporation, Tokyo, Japan).

### Statistical analysis

Data were analyzed using Graphpad®Prism® version 5.04 (GraphPad Software Inc., La Jolla, USA). Comparisons between groups were performed by unpaired Student's t-test. A two-sided *P* value <.05 was considered statistically significant.
